# Zearalenone exposure affects the Wnt/β-catenin signaling pathway and related genes of porcine endometrial epithelial cells *in vitro*

**DOI:** 10.5713/ajas.20.0292

**Published:** 2020-08-24

**Authors:** Tingting Song, Weiren Yang, Libo Huang, Zaibin Yang, Shuzhen Jiang

**Affiliations:** 1 Department of Animal Sciences and Technology and Shandong Provincial Key Laboratory of Animal Biotechnology and Disease Control and Prevention, Shandong Agricultural University, Taian, Shandong, 271 018, China

**Keywords:** Zearalenone, Porcine Endometrial Epithelial Cells, WNT1, β-Catenin, GSK-3β, Cyclin D1

## Abstract

**Objective:**

Zearalenone (ZEA) has estrogen-like effects. Our previous study has shown that ZEA (0.5 to 1.5 mg/kg) could induce abnormal uterine proliferation through transforming growth factor signaling pathway. To further study the other regulatory networks of uterine hypertrophy caused by ZEA, the potential mechanism of ZEA on porcine endometrial epithelial cells (PECs) was explored by the Illumina Hiseq 2000 sequencing system.

**Methods:**

The PECs were treated with ZEA at 0 (ZEA0), 5 (ZEA5), 20 (ZEA20), and 80 (ZEA80) μmol/L for 24 h. The collected cells were subjected to cell cycle, RNA-seq, real-time quantitative polymerase chain reaction, immunofluorescence, and western blot analysis.

**Results:**

The proportion of cells in the S and G2 phases decreased (p<0.05), but the proportion of cells in the G1 phase increased (p<0.05) in the ZEA80 treatment. Data analysis revealed that the expression of Wnt pathway-related genes, estrogen-related genes, and mitogen-activated protein kinase pathway-related genes increased (p<0.05), but the expression of genetic stability genes decreased (p<0.05) with increasing ZEA concentrations. The relative mRNA and protein expression of WNT1, β-catenin, glycogen synthase kinase 3β (GSK-3β) were increased (p<0.05) with ZEA increasing, while the relative mRNA and protein expression of cyclin D1 (CCND1) was decreased (p<0.05). Moreover, our immunofluorescence results indicate that β-catenin accumulated around the nucleus from the cell membrane and cytoplasm with increasing ZEA concentrations.

**Conclusion:**

In summary, ZEA can activate the Wnt/β-catenin signaling pathway by up-regulating WNT1 and β-catenin expression, to promote the proliferation and development of PECs. At the same time, the up-regulation of GSK-3β and down-regulation of CCND1, as well as the mRNA expression of other pathway related genes indicated that other potential effects of ZEA on the uterine development need further study.

## INTRODUCTION

Zearalenone (ZEA), also known as F-2 toxin, is a nonsteroidal estrogenic mycotoxin with the formula C_18_H_22_O_5_, and is produced by several strains of filamentous fungi in the genus *Fusarium* [1]. Fungi in the genus *Fusarium* are present in cereals such as barley, sorghum, oats, maize, and wheat [2]. ZEA can endanger human health directly, via contaminated food, as well as indirectly, through contaminated animal products [3]. ZEA, as well as its main metabolites, α-zearalanol (α-ZOL) and β-zearalanol (β-ZOL), have been shown to have estrogenic activity owing to their structural similarity to 17β-estradiol [4–6]. ZEA can lead to a range of reproductive disorders, such as ovarian abnormalities, pseudopregnancy, infertility, and miscarriage [7].

Our previous studies showed that ZEA at dietary concentrations ranging from 1.1 to 3.2 mg/kg increased hyperplasia of submucosal smooth muscles in the uteri of gilts [8], and, at concentrations of 0.5 to 1.5 mg/kg, ZEA increased the uterine organ index and the thickness of the myometrium and endometrium [9,10]. However, the relationship between the uterine cells changes and uterine hypertrophy induced by ZEA has not been fully elucidated. In the present study, the porcine endometrial epithelial cells (PECs) were selected to initially explore the potential mechanisms of ZEA on the uterine hypertrophy through the Wnt pathway and related genes, and our next subject is endometrial stromal cells and smooth muscle cells, respectively.

The Wnt/β-catenin signaling pathway has been shown to be responsible for numerous fundamental processes essential for embryonic development, normal adult homeostasis, and cancer [11]. It was reported that the promotion of hair growth was often observed upon treatment with Wnt agonists [12]. Moreover, Lee et al [13] indicated that the activated Wnt signaling pathway could promote the proliferation of human dermal papilla in dermal papilla cells. The core Wnt/β-catenin signaling pathway components, such as Wnt, glycogen synthase kinase 3β (GSK-3β), β-catenin, and transcription factors (TCF) control the life cycle of Wnt signaling molecules and the actions of target cells. The target gene cyclin D1 (CCND1) of the pathway is the regulator of the G1/S phase transition in the cell cycle. In addition, mitogen-activated protein kinase (MAPK) and transforming growth factor (TGF) signaling pathways have been reported to act on the Wnt pathway involved in cell growth and development [14]. The Wnt signaling pathway plays an important role in animal growth and development. However, there is little information about the relationship between ZEA-induced uterine hypertrophy and the Wnt signaling pathway. Therefore, the objective of this study was to explore the potential mechanisms behind the effects of ZEA on the growth and development of PECs by the Wnt pathway and related genes.

## MATERIALS AND METHODS

### Preparation of PECs culture

The PECs were donated by the Laboratory of Metabolic Diseases of Shandong Agricultural University, which has kept homogeneous population and invariant properties after more than 50 generations [15]. For each assay, the cell cryotube containing PECs was removed from the liquid nitrogen and immediately placed in a 37°C water bath for 1.5 min, and then the outside of the tube was sterilized with 70% ethanol before placing the tube into an ultra-clean platform. The cell content in the tube was then transferred into a 1.5 mL centrifuge tube and centrifuged at 1,000×*g* for 3 min. After removal of the supernatant, the PECs were cultured with 2 mL of DMEM/F-12 (01-172-1ACS, Biological Industries, Kibbutz Beit-Haemek, Israel) containing 10% fetal bovine serum (FBS, FS101-02, TRAN, Beijing, China). The incubation was conducted at 37°C in a cell incubator containing 5% CO_2_ before use in each assay, as described below.

### Preparation of ZEA and ZEA treatment of PECs

ZEA was obtained commercially (Z2125; Sigma, St. Louis, MO, USA), and was dissolved in dimethyl sulfoxide (DMSO) (D2650; Sigma, USA) at a concentration of 20 mmol/L, and was stored at −20°C before use.

For each assay conducted below, the PECs prepared above were first distributed into 6-well plates (1×10^6^ cells per well), to which aliquots of ZEA solution were added so that the concentrations of ZEA in the culture reached 0 (Control), 5 (ZEA5), 20 (ZEA20), 40 (ZEA40), and 80 (ZEA80) μmol/L, respectively. In order to eliminate the influence of DMSO among treatments, the DMSO of all treatment was adjusted to the level of ZEA80. After mixing, the cultures were incubated at 37°C for 24 h, and then the cells were harvested and processed according to the descriptions for each assay. Each treatment was repeated at least three times, in the experiment.

### Determination of the effects of ZEA on cell cycle distribution of PECs

A flow cytometry assay was conducted to determine the effects of ZEA on the cell cycle distribution of PECs according to the kit procedure and Zhang et al [15]. At the end of the incubation with varying concentrations of ZEA as described above, the PECs were collected using 0.25% trypsin, centrifuged to remove trypsin, washed twice with 2 mL cold phosphate-buffered saline (PBS), and re-suspended in cold 70% ethyl alcohol. The suspension was stored at 4°C for overnight, and was subsequently centrifuged at 1,000×*g* for 5 min, followed by two cycles of washing with 2 mL cold PBS and centrifugation (1,000×*g*, 5 min). The harvested PECs were then stained with propidium iodide (C1052-2; Beyotime, Shanghai, China) and RNase A (C1052-3; Beyotime, China) at 37°C in the dark for 30 min, and subsequently analyzed using a BD FACSCalibur flow cytometer (FACSCalibur, BD, San Jose, CA, USA) using FlowJo 7.6 software. The cell cycle stages of PECs were defined as G1, S, and G2, which were identified as described by Zhang et al [15].

### Determination of the effects of ZEA on the Wnt signaling pathway, and on relative mRNA expression of Wnt pathway related genes

At the end of the 24-h ZEA treatment described above, the PECs were obtained (as described above). Total RNA was extracted from the cells using RNAiso Plus (Applied TaKaRa, Dalian, China) according to the manufacturer’s instructions. The purity and concentration of the RNA were determined using an Eppendorf Biophotometer (DS-11, Denovix, Wilmington, DE, USA) at an absorbance ratio of 260/280 nm. Total RNA was reverse transcribed to cDNA using a Reverse Transcription System Kit (PrimeScript RT Master Mix, RR036A, Applied TaKaRa, China), and the resultant cDNA was divided into two subsamples. cDNA was stored at −20°C until it was used for transcriptome sequencing and real-time quantitative polymerase chain reaction (RT-qPCR) quantification.

One cDNA subsample was sequenced at Novogene Co. (Beijing, China) using the Illumina Hiseq 2000 sequencing system (San Diego, CA, USA), and the data obtained were aligned to the reference genome using HISAT2 software, with the image data converted into reads by CASAVA base recognition. Based on the positional information of the gene on the reference genome, the number of reads covered by each of the entire genes was counted, which was then used to quantify the amount of gene expression using ‘featureCounts’ in the Subread software.

The other subsample of cDNA was used for RT-qPCR quantification. The reaction mixture contained 10 μL SYBR, 0.4 μL Dye II, 1.9 μL forward and reverse primers, 6 μL nuclease-free water, and 2 μL cDNA. The nucleotide sequences of all genes investigated in the GD region were obtained from the Gene Bank database (https://www.ncbi.nlm.nih.gov/). All primers were designed by Sangon Biological Engineering Technology and Services Co. Ltd. (Shanghai, China) and synthesized by the Beijing Genomics Institute (BGI, Beijing, China). The primer sequences and product lengths are presented in Table 1. The optimized RT-qPCR protocol included an initial denaturation step at 95°C for 30 s, followed by 43 cycles at 95°C for 5 s, 60°C for 34 s, 95°C for 15 s, 60°C for 60 s, and 95°C for 15 s. The qRT-PCR reaction was conducted in a Light Cycler 96 (LC96, Roche, Germany).

### Determination of the effect of ZEA on β-catenin distribution in PECs

The PECs were first seeded on microslides, which were then subjected to ZEA treatments as described above, and subsequently fixed with 4% paraformaldehyde for 1 h and permeabilized with Triton X-100 (0.5%) for 10 min at room temperature. The resultant cells were processed using the following steps: washing with PBS three times for 5 min each, blocking with 10% FBS for 1 h, incubating with anti-β-catenin (1:100, sc-7199, Santa Cruz Biotechnology, Shanghai, China) at 4°C overnight, washing with PBS three times, mixing with diluted Alexa Fluor 555-labeled donkey anti-rabbit immunoglobulin G (lgG) (1:500, A0453, Beyotime, China) at 37°C in the dark for 1 h, and washing with PBS. The processed cells were subsequently treated with the appropriate Hoechst 33342 nucleic acid stain (C1022, Beyotime, China), mixed for 5 min, and then washed with PBS. The processed samples were examined under a confocal microscope (FLUOVIEW FV3000, Olympus, Tokyo, Japan).

### Determination of the effects of ZEA on protein expression of WNT1, β-catenin, GSK-3β, and CCND1

After 24 h incubation with ZEA, 500 μL of 0.25% trypsin was added to the culture, and the culture was centrifuged at 1,200×*g* for 5 min. The resultant pellet was rinsed with 2 mL PBS and centrifuged again (1,200×*g*, 5 min), which was subsequently extracted with lysate containing phenylmethanesulfonyl fluoride (1 mmol/L, Beyotime, China) according to the manufacturer’s protocol. The extract was first analyzed for total protein content using a BCA protein assay kit (Beyotime, China), and was then subjected to western blotting to determine the protein expression of relevant mRNAs as described below. Each sample containing 60 μg of protein was loaded on polyacrylamide gels and subjected to electrophoresis for 1.5 h. The separated bands were then transferred to immobilon-p transfer membranes (Solarbio, Beijing, China), which were first blocked in 10% skimmed milk powder for 2 h, washed with Tris buffered saline Tween (TBST, pH 7.6) three times, and then incubated with monoclonal mouse antibody β-actin (1:5,000, ab8226, Abcam, Cambridge, UK), monoclonal mouse antibody WNT1 (1:200, ab105740, Abcam, UK), polyclonal rabbit antibody β-catenin (1:5,000, ab6302, Abcam, UK), monoclonal mouse antibody GSK-3β (1:1,000, ab93926, Abcam, UK), and monoclonal rabbit antibody CCND1 (1:100, ab40754, Abcam, UK) overnight at 4°C. After incubation, the membrane was washed with TBST three times for 5 min each, and further incubated with anti-rabbit lgG (1:5,000, ab6721, Abcam, UK) and anti-mouse lgG (1:5,000, ab6728, Abcam, UK) in diluted secondary antibody dilution buffer (Beyotime, China) at room temperature for 2 h, followed by washing with TBST (three times for 5 min each). The resultant membrane was then immersed in a high-sensitivity luminescence reagent (BeyoECL Plus, Beyotime, China) and exposed to film using FusionCapt Advance FX7 (Beijing Oriental Science and Technology Development Co. Ltd., Beijing, China) to determine the gray bands labeled by the target protein. BandScan 5.0 software was used to analyze the optical density values. Each band was repeated at least three times. The relative expression of the target protein was target protein (optical density value)/internal reference (optical density value).

### Statistical and analysis

All data were analyzed using the general linear model procedure of SAS 9.2 (SAS Institute Inc., Cary, NC, USA). Analysis of variance was performed by one-way analysis. Orthogonal polynomial contrasts were used to determine linear responses to the ZEA treatments. Significant differences among treatments were further analyzed using Tukey’s HSD test. All statements of significance were based on a probability of p<0.05.

## RESULTS

### Effects of ZEA on the cell cycle distribution of PECs

**Figure 1:**
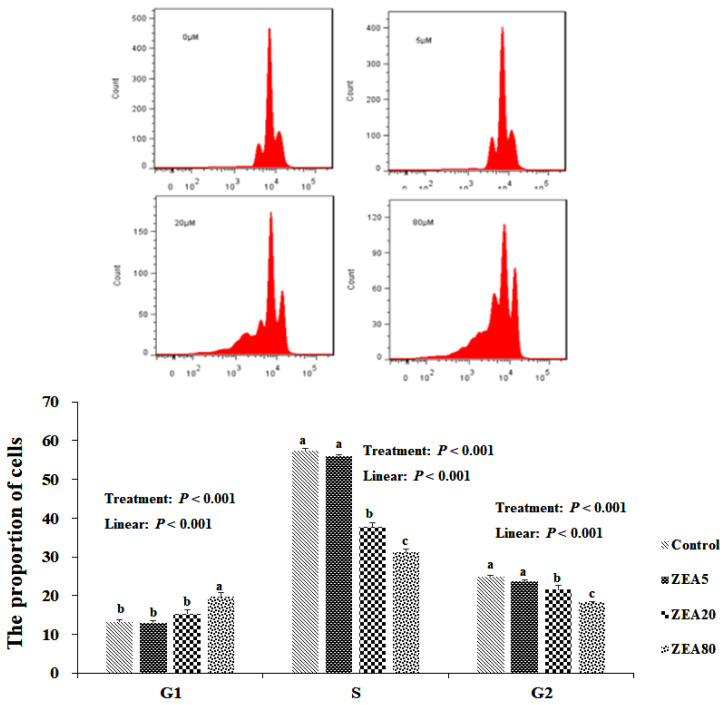
Effect of zearalenone (ZEA) on the cell cycle of porcine endometrial epithelial cells (PECs) exposed to ZEA at 0 (Control), 5 (ZEA5), 20 (ZEA20), and 80 (ZEA80) μmol/L for 24 h. Cell cycle of PECs determined by flow cytometer after propidium iodide (PI). ^a–c^ Values within a column with the different letters mean significantly different (p<0.05). The p-values of treatment and linear were obtained from one-way analysis of variance and orthogonal polynomial contrasts, respectively. All experiments were repeated three times.

Cell cycle analysis by flow cytometry showed that, compared with the control treatment, incubating PECs with ZEA at a concentration of 80 μmol/L significantly increased (p<0.05) the proportion of cells in G1 phase, but decreased (p<0.05) the proportions of cells in the S and G2 phases (Figure 1). A decrease in the proportion of cells in S and G2 phases was also observed at a ZEA concentration of 20 μmol/L.

### Effects of ZEA on the Wnt signaling pathway and on relative mRNA expression of Wnt pathway-related genes

**Figure 2:**
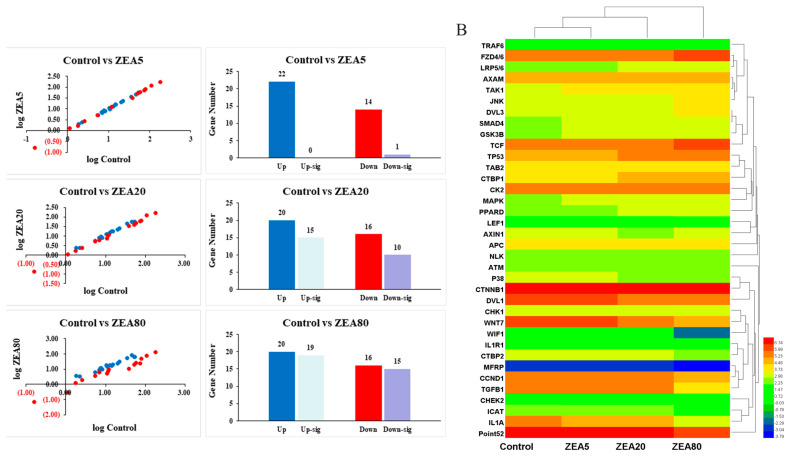
Screening analysis of mRNA on Wnt/β-catenin signaling pathway relative genes of the porcine endometrial epithelial cells (PECs) exposed to ZEA at 0 (Control), 5 (ZEA5), 20 (ZEA20), and 80 (ZEA80) μmol/L for 24 h. (A) Relative mRNA expression analysis by scatter plot and histogram. The red spots indicate up-regulated genes, and the blue spots indicate down-regulated genes. The histogram detailed analysis on the up or down regulated genes. Up-sig, significantly up-regulated (p<0.05). Down-sig, significantly down-regulated (p<0.05). (B) Hierarchical clustering analysis (heatmap) for genes using Pearson’s correlation. The p-values of treatment and linear were obtained from one-way analysis of variance and orthogonal polynomial contrasts, respectively.

Thirty-six Wnt pathway-related genes were screened by RNA-seq (Figure 2B). Compared with the control treatment, treating PECs with treatments ZEA20 and ZEA80 altered expression of 25 and 34 genes of the 36 identified genes, respectively. Further analysis showed that 15 and 19 genes were upregulated (p<0.05) and 10 and 15 genes were downregulated (p< 0.05) by ZEA20 and ZEA80 treatments, respectively (Figure 2A). In contrast, compared with the control treatment, only one gene was downregulated by the ZEA5 treatment.

**Figure 3:**
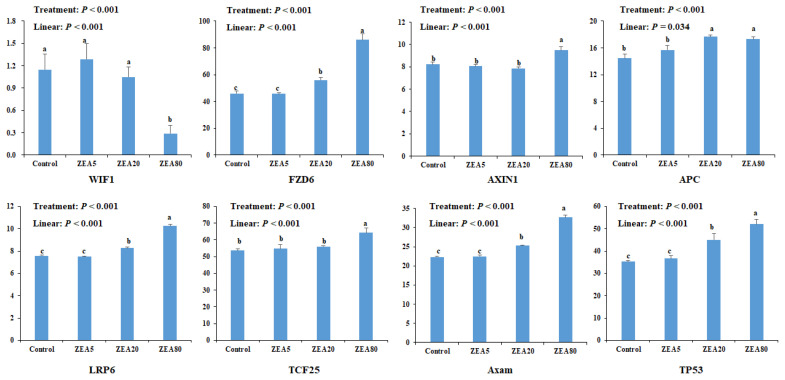
The mRNA expression of Wnt/β-catenin signaling pathway related genes in porcine endometrial epithelial cells (PECs) exposed to ZEA at 0 (Control), 5 (ZEA5), 20 (ZEA20), and 80 (ZEA80) μmol/L for 24 h by RNA-seq. *WIF1*, Wnt inhibitory factor 1; *FZD6*, Frizzled 6, *AXIN1*, Axin1; APC, adenomatous polyposis coli; *LRP6*, low density lipoprotein receptor related protein 6, *TCF25*, transforming growth factors 25; *TP53*, tumor protein 53. ^a–c^ Values with a column with the different letters mean significantly different (p<0.05). The p-values of treatment and linear were obtained from one-way analysis of variance and orthogonal polynomial contrasts, respectively. All experiments were repeated 3 times.

Eight genes were selected for quantitative analysis in rela tion to the ZEA treatments (Figure 3). The expression of Frizzled 6 (FZD6), AXIN1, adenomatous polyposis coli (APC), Axam, TCF25, low density lipoprotein receptor related protein 6 (LRP6), and tumor protein 53 (TP53) increased linearly (p<0.05) with increased ZEA concentrations. However, expression of Wnt inhibitory factor 1 (WIF1) decreased linearly (p<0.05) with increasing ZEA concentrations. The mRNA expression of FAD6, APC, Axam, and TP53 in the ZEA20 and ZEA80 treatments, and the mRNA expression of AXIN1, LRP6, and TCF25 in the ZEA80 treatment were higher (p< 0.05) than those in the control treatment, but the mRNA expression of WIF1 in the ZEA80 treatment was lower (p<0.05) than that in the control treatment.

### Effects of ZEA on relative mRNA expression of other pathway-related genes

**Figure 4:**
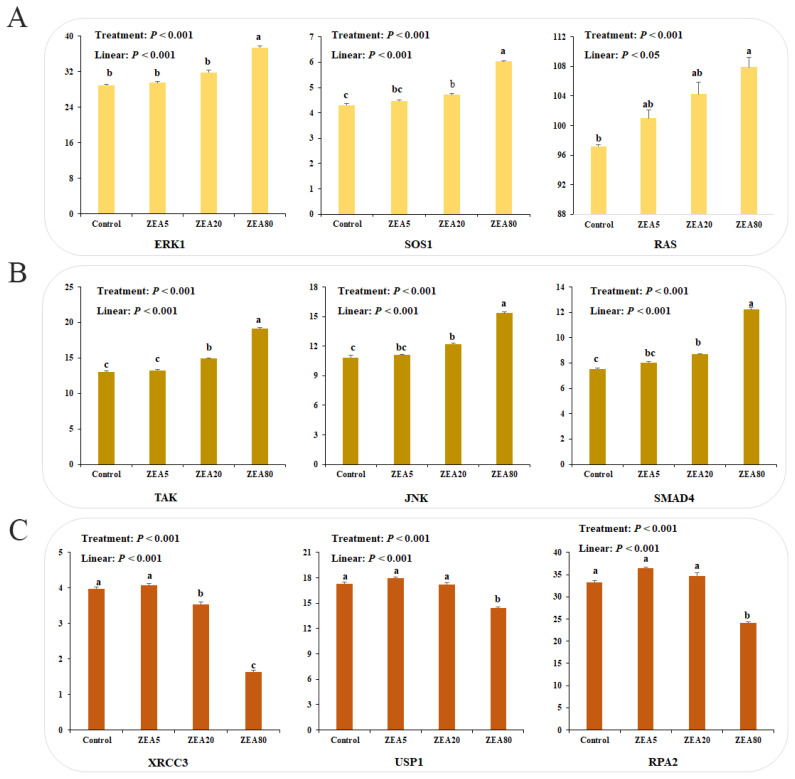
The mRNA expression of other related pathway genes in porcine endometrial epithelial cells (PECs) exposed to ZEA at 0 (Control), 5 (ZEA5), 20 (ZEA20), and 80 (ZEA80) μmol/L for 24 h by RNA-seq. (A) Estrogen signaling pathway related genes. (B) MAPK and TGF signaling pathway related genes. (C) Genetic stability related genes. *ERK1*, extracellular signal-regulated kinase 1; *SOS1*, SOS Ras/Rac guanine nucleotide exchange factor 1; JNK, c-Jun N-terminal kinase; *TAK*, TGF-beta activated kinase; *SMAD4*, SMAD family member 4; *XRCC3*, X-ray repair cross complementing 3; *USP1*, ubiquitin specific peptidase 1; *RPA2*, replication protein A2. ^a–c^ Values with a column with the different letters mean significantly different (p<0.05). The p-values of treatment and linear were obtained from one-way analysis of variance and orthogonal polynomial contrasts, respectively. All experiments were repeated 3 times.

RNA-seq results indicate that the mRNA expression of estrogen-related genes, including extracellular signal-regulated kinase 1 (*ERK1*), SOS Ras/Rac guanine nucleotide exchange factor 1 (*SOS1*), Ras, *MAPK*-related genes, including TGF-beta activated kinase (*TAK*) and c-Jun N-terminal kinase (*JNK*), and TGF-related genes, including SMAD family member 4 (*SMAD4*), increased linearly with increasing ZEA concentrations (p<0.05). However, mRNA expression of genetic stability genes such as X-ray repair cross complementing 3 (*XRCC3*), ubiquitin specific peptidase 1 (*USP1*), and replication protein A2 (*RPA2*) decreased linearly (p< 0.05) with increasing ZEA concentrations (Figure 4). The mRNA expression levels of SOS1, TAK, JNK, and SMAD4 in the ZEA20 and ZEA80 treatments, and mRNA expression levels of ERK1 and RAS in the ZEA80 treatment were higher (p<0.05) than those in other treatments. The mRNA expression levels of XRCC3 in the ZEA20 and ZEA80 treatments, and of USP1 and RPA2 in the ZEA80 treatment, were lower (p<0.05) than those in the control treatment.

### Comparative analysis of relative mRNA expression by RNA-seq and qRT-PCR after ZEA exposure

In order to verify the accuracy of RNA-seq, WNT1, β-catenin, GSK-3β, and CCND1 were selected for analysis by qRT-PCR (Table 2). The relative mRNA expression levels of WNT1, β-catenin, and GSK-3β in the PECs by qRT-PCR and RNA-seq analysis all increased linearly (p<0.05) with increasing ZEA concentrations, but the mRNA expression levels of CCND1 decreased linearly (p<0.05) with increasing ZEA concentrations. The mRNA expression levels of WNT1, β-catenin, and GSK-3β in the ZEA80 treatment were higher (p<0.05) than those in the control and ZEA5 treatments, and mRNA expression levels of CCND1 in the ZEA80 treatment was lower (p<0.05) than that in the control and ZEA5 treatments in both qRT-PCR and RNA-seq analyses.

### Effects of ZEA on the immunofluorescence localization of β-catenin in PEGs

**Figure 5:**
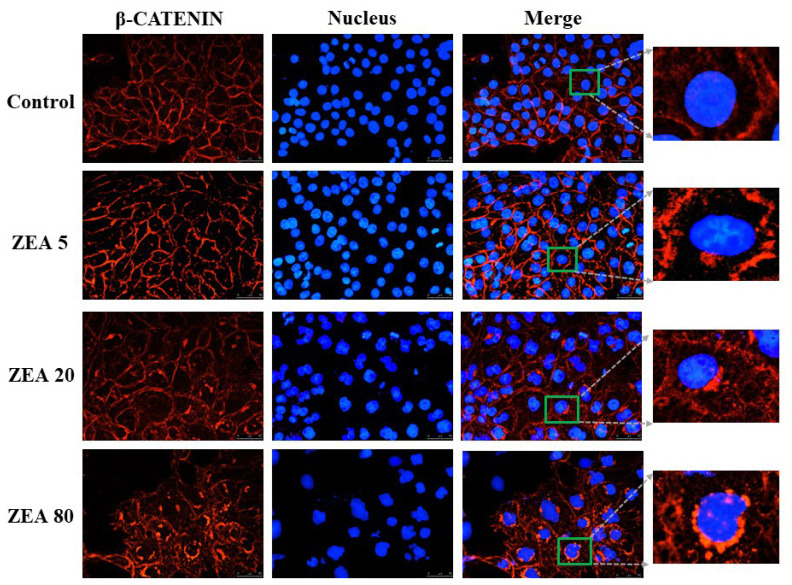
Immunostaining of β-catenin of porcine endometrial epithelial cells (PECs) exposed to ZEA at 0 (Control), 5 (ZEA5), 20 (ZEA20), and 80 (ZEA80) μmol/L for 24 h, and stained by indirect immunofluorescence and observed under a light microscope (40×).

The immunofluorescence result of β-catenin is shown in Figure 5. In the control treatment, β-catenin was mainly distributed in the cell membrane, with a small amount distributed in the cytoplasm. As the ZEA concentration increased, β-catenin aggregated around the nucleus, and the immunopositivity in the cytoplasm was enhanced. The immunoreactive substance of β-catenin was the strongest around the nucleus and it showed signs of entering the nucleus in the ZEA80 treatment.

### Effects of ZEA treatment on expression of WNT1, β-catenin, GSK-3β, and CCND1 proteins

**Figure 6:**
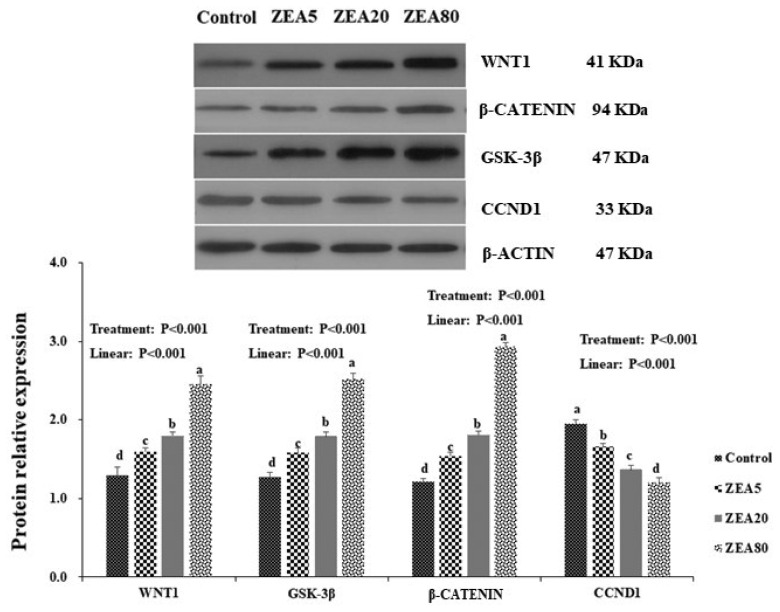
Western blot analysis of Wnt1, β-catenin, glycogen synthaes kinase 3β (GSK-3β), Cyclin D1 (CCND1) of the porcine endometrial epithelial cells (PECs) exposed to ZEA at 0 (Control), 5 (ZEA5), 20 (ZEA20), and 80 (ZEA80) μmol/L for 24 h. ^a–d^ Values within a column with the different letters mean significantly different (p<0.05). The p-values of treatment and linear were obtained from one-way analysis of variance and orthogonal polynomial contrasts, respectively. All experiments were repeated at least three times.

Western blot analysis revealed positive bands of appropriate sizes for WNT1, β-catenin, GSK-3β, CCND1, and β-actin genes (Figure 6). The antibodies were detected as single bands at 41, 94, 37, 33, and 42 kDa for the respective genes. The relative protein expression of WNT1, β-catenin, and GSK-3β in the PECs increased linearly (p<0.05) with increasing concentrations of ZEA. However, the protein expression of CCND1 decreased linearly (p<0.05) as the ZEA concentration increased. In general, the protein expressions of WNT1, β-catenin, and GSK-3β increased with increasing ZEA concentrations, and were ranked in the following order: ZEA80> ZEA20>ZEA5>control (p<0.05). The protein expression of CCND1 decreased with increasing ZEA concentrations, in the following order: control>ZEA5>ZEA20>ZEA80 (p<0.05).

## DISCUSSION

### Effect of ZEA on the Wnt signaling pathway and related genes of PECs

The observation that ZEA promotes the expression of the Wnt/β-catenin signaling pathway and related genes of PECs *in vitro* in the current study is consistent with the results of Yang et al [16]. The Wnt/β-catenin signaling pathway participates in various biological processes, and its hyperactivation is closely related to cancer development and progression. It has already been shown that the Wnt/β-catenin signaling pathway is involved in many cellular functions that are essential for normal organ development, such as cell proliferation, survival, and self-renewal. It was reported that, in the absence of Wnt signal stimulation, the proto-oncoprotein β-catenin is bound by a multiprotein complex consisting of the protein APC, Axin, and GSK-3β. In this complex, β-catenin is phosphorylated and is therefore marked for ubiquitination via β-transducin repeat containing protein and subsequent proteasomal degradation [17]. In cells with an active pathway, the complex is destabilized; β-catenin is no longer bound and accumulates in the cytoplasm and then translocates into the nucleus. In the nucleus, β-catenin binds to the lymphoid enhancer factor/T-cell factor to initiate the transcription of target genes that promote the proliferation, migration, and invasion of cancer cells [18]. Our present study indicated that ZEA altered the mRNA expression of WNT1, β-catenin, GSK-3β, and CCND1 involved in the Wnt signaling pathway. Moreover, more significant changes in gene expression occurred as the ZEA concentration increased. This suggests that ZEA may activate the Wnt signaling pathway by altering the expression of genes involved in the Wnt signaling pathway, thereby promoting cell proliferation. WNT1 is the initiating factor of the canonical Wnt/β-catenin signaling pathway and is highly expressed in many malignant tumors. One of the most important functions of WNT1 is to inhibit cell apoptosis by increasing the invasiveness of cells and promoting the growth of blood vessels, thereby participating in tumor formation. It was reported that β-catenin is the key effector of the Wnt signaling pathway [19], and is also an essential factor that transmits signals to the nucleus, initiates transcription of Wnt-specific genes, and determines the specificity of various cells and tissues [20]. Immunofluorescence results showed that β-catenin gradually moved from the cell membrane and cytoplasm to the nucleus with increases in ZEA concentration, and showed signs of entering the nucleus, which proves that the Wnt pathway was indeed activated. Therefore, ZEA induced the high expression of WNT1, which led to the accumulation of β-catenin in the cytoplasm and finally into the nucleus, activated Wnt/β-catenin pathway, and eventually led to cell proliferation.

A large number of studies have shown that GSK-3β acts as the key negative regulator of the Wnt/β-catenin signaling pathway [18,21]. Studies have shown that the Wnt pathway can be activated by inhibiting the activity of GSK-3β [19]. Moreover, some Wnt agonists have been shown to target GSK-3β, but the exact binding sites are not fully clear. The results that mRNA and protein expression levels of GSK-3β increased linearly with increasing concentrations of ZEA in this study were contrary to the results of a previous study [22], but were consistent with findings of Yang et al [16] who showed that the expression of GSK-3β increased with increasing concentrations of ZEA in the ovaries. Li et al [23] reported that the overexpression of GSK-3β promoted the overexpression of WISP-1, which in turn inhibited Caspase-3 expression and led to attenuated tumor cell apoptosis and promoted tumor development. Therefore, the overexpression of GSK-3β may be an important reason for cell proliferation when Wnt pathway is activated However, the mechanism by which GSK-3β regulates the Wnt pathway in PECs needs to be validated using an inhibitor test.

It has been reported that CCND1 is an important target gene in the Wnt signaling pathway, and its abnormal expression is correlated with activation of the pathway [24]. CCND1 is one of the main regulators of the G1 to S phase transition during the proliferative stage of the cell cycle. The observation that expression of CCND1 decreased with increasing ZEA concentrations corresponds with the results that incubation with ZEA caused cell cycle arrest in the G1 phase in this study. Previous study has shown that GSK-3β negatively regulated CCND1 through enhancing proteasomal degradation of CCND1 by phosphorylating its Thr286 and inhibiting *CCND1* gene transcription by increasing degradation of β-catenin [25]. These results suggest that ZEA promoted high expression of GSK-3β, which suppressed the expression of CCND1, thereby disrupting the cell cycle. However, cell cycle arrest often leads to apoptosis. But the result of this study showed that 5 to 80 μmol/L ZEA activated the Wnt pathway while causing cell cycle arrest. Which indicated that ZEA could promote proliferation and development of PECs by activating or overexpressing Wnt signaling pathway, and might cause cell apoptosis. Therefore, ZEA has a more complex mechanism for cell reproductive toxicity, which need to further study.

### Effect of ZEA on other signaling pathways and related genes of PECs

Studies have reported that a low dose of ZEA could exert estrogen-like effects and exhibit carcinogenic properties, which stimulate the proliferation of cells [26]. Estrogen receptors can activate cell signaling pathways, and ERK1/2 mediates the estrogen-like signal for cell proliferation, a mechanism by which estrogen regulates biological processes [27]. The mRNA expression of ERK1 after ZEA treatment (20 and 80 μmol/L) was significantly higher than that in the control treatment in the present study. Moreover, the mRNA expression of the upstream genes of ERK1, such as SOS1 and RAS, was significantly upregulated in the ZEA treatments, especially in the ZEA80 treatment. The MAPK signaling pathway is closely linked to the estrogen pathway and plays an important role in promoting cell growth and cancer development [28]. The mRNA expression of MAPK pathway-related genes *TAK* and *JNK* increased significantly with increasing ZEA concentrations. The results indicate that ZEA was able to alter the expression of genes involved in growth and development, which may promote cell proliferation. Research has also shown that ZEA can cause DNA damage and induce chromosome aberrations, which might be an important mechanism for inducing cancer [26]. However, the cell cycle checkpoint can block or delay cell cycle progression to repair the damaged DNA or regulate cell apoptosis in situations of DNA damage, mutations, or other abnormal conditions [29]. The observation that the mRNA expression levels of XRCC3, USP1, and RPA2 were significantly reduced with increasing ZEA concentrations, however, the effects of ZEA on cell migration, mitochondrial metabolism, cell differentiation inhibition and DNA repair related genes need to be further confirmed. Liu et al [30] also reported similar results. This also was consistent with the result that ZEA, at concentrations of 20 and 80 μmol/L, caused a significant reduction in the proportion of cells in the S phase of the cell cycle, leading to G1/G0 arrest. Liu et al [30] demonstrated that ZEA could impair the genomic stability of swine follicular granulosa cells. Based on this information, it was suggested that ZEA could disrupt the cell cycle of PECs by damaging DNA, which might lead to cancer and promote cell proliferation. Therefore, ZEA promotes proliferation of PECs, possibly through estrogen-like effects and carcinogenesis, but the molecular mechanisms behind this need further study.

### Proposed model of Wnt/β-catenin pathways in response to ZEA exposure in PECs

**Figure 7:**
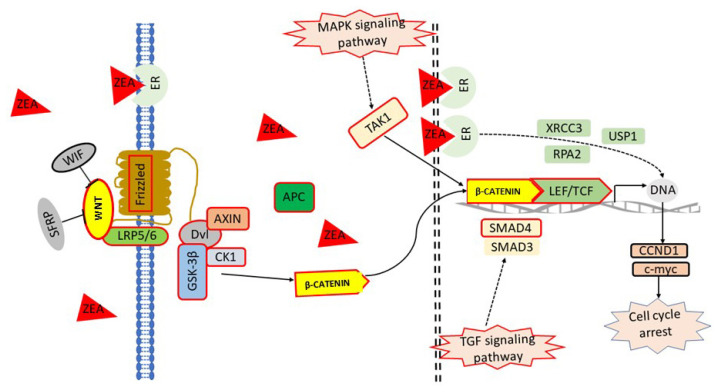
The signal pathways map of Wnt/β-catenin. Arrows indicate activation/induction, blunt ended lines indicate inhibition/blockade. Solid arrows indicate direct action, dotted arrows indicate indirect action.

Based on the information obtained above, a model that the ZEA on the Wnt/β-catenin centered regulatory network of PCEs was proposed (Figure 7). When PCEs were exposed to ZEA, extracellular Wnt was stimulated. The trimers of GSK-3β, Axin, and APC were unstable, which induced β-catenin to escape from ubiquitin hydrolysis system and accumulate in the cytoplasm. Meanwhile, MAPK pathway was activated by estrogen receptor pathway, which promoted the transfer of β-catenin from cytoplasm to the nucleus. Eventually, the nuclear TGF pathway activated by ZEA strengthened the binding of β-catenin to TCF/LEF family transcription factors in the nucleus and initiated downstream transcription.

## CONCLUSION

In summary, ZEA can promote the growth and development of PECs by altering the expression of the Wnt/β-catenin signaling pathway and related genes. The accumulation of β-catenin in the nucleus and the expression of WNT1 indicate that ZEA activates the Wnt signaling pathway. However, the increase in expression levels of GSK-3β and the decrease in expression levels of CCND1 in the PECs indicated a more complex relationship among the pathways. Therefore, further studies are needed to explore the underlying mechanisms using gene blocking tests.

## Figures and Tables

**Table 1:** Primer sequences of target genes

Target genes	Primer sequence (5′-3′)	Product size (bp)
*GAPDH*	F: ATGGTGAAGGTCGGAGTGAA R: CGTGGGTGGAATCATACTGG	154
*WNT1*	F: GGAACCGCCGCTGGAATTGTC R: CGCCGCCTATAGTCGCATGTG	181
*β-catenin*	F: GACCATGCCATGATTGGACCTGAG R: GCCTGTCAACCTTCTCGCTGTC	166
*GSK-3β*	F: CAGAGACAAGGATGGCAGCAAGG R: TGGCAACCAGTTCTCCTGAATCAC	164
*CCND1*	F: CCTGTCCTACCACCGCCTGAC R: ACCTCCTCCTCTTCCTCCTCCTC	174

*GAPDH*, glyceraldehyde-3-phosphate dehydrogenase; *GSK-3β*, glycogen synthaes kinase 3β; *CCND1*, cyclin D1.

**Table 2:** Effect of zearalenone on the relative mRNA expression of Wnt1, β-catenin, glycogen synthaes kinase 3β (GSK-3β) and cyclin D1 (CCND1) in the porcine endometrial epithelial cells

Items		WNT1		β-catenin		GSK-3β		CCND1	
			
		RNA-seq	qRT-PCR	RNA-seq	qRT-PCR	RNA-seq	qRT-PCR	RNA-seq	qRT-PCR
Control1)		1.00^d^	1.00^b^	1.00^d^	1.00^c^	1.00^c^	1.00^c^	1.00^a^	1.00^a^
ZEA51)		2.46^c^	1.04^b^	1.18^c^	1.08^bc^	1.06^bc^	1.08^bc^	0.98^a^	0.78^b^
ZEA201)		2.90^b^	2.32^a^	1.27^b^	1.26^ab^	1.14^b^	1.22^b^	0.82^b^	0.68^bc^
ZEA801)		3.11^a^	2.89^a^	1.34^a^	1.34^a^	1.46^a^	1.54^a^	0.44^c^	0.59^c^
SEM		0.249	0.194	0.039	0.037	0.055	0.049	0.068	0.047
p-values	Treatment	<0.001	<0.001	<0.001	<0.001	<0.001	<0.001	<0.001	<0.001
	Linear	<0.001	<0.001	<0.001	<0.001	<0.001	<0.001	<0.001	<0.001

GSK-3β, glycogen synthaes kinase 3β; CCND1, cyclin D1; qRT-PCR, real-time quantitative polymerase chain reaction; SEM, standard error of the mean.

1) Control, ZEA5, ZEA20 and ZEA80 were porcine endometrial epithelial cells (PECs) exposed to ZEA at 0, 5, 20 and 80 μmol/L for 24 h.

a–d Values within a column with the different letters mean significantly different (p<0.05). The p-values of treatment and linear were obtained from one-way analysis of variance and orthogonal polynomial contrasts, respectively.
